# Supplementary material for: Gender equality related to gender differences in life expectancy across the globe gender equality and life expectancy
Source: PLOS Glob Public Health. 2023 Mar 6;3(3):e0001214. doi: 10.1371/journal.pgph.0001214 (PMC10021358; doi:10.1371/journal.pgph.0001214)
Supplement: S1 Table — (DOCX) [file pgph.0001214.s001.docx]

**S1 Table: Subindexes included in the Global Gender Gap Index**

| Subindex | Indicator |
| --- | --- |
| Economic Participation and Opportunity | Labour force participation rate (%) |
|  | Wage equality for similar work (survey, 1–7 scale) |
|  | Estimated earned income (PPP, int.$) |
|  | Legislators, senior officials and managers (%) |
|  | Professional and technical workers (%) |
| Educational Attainment | Literacy rate (%) |
|  | Enrolment in primary education (%) |
|  | Enrolment in secondary education (%) |
|  | Enrolment in tertiary education (%) |
| Health and Survival | Sex ratio at birth (%) |
|  | Healthy life expectancy (years) |
| Political Empowerment | Women in parliament (%) |
|  | Women in ministerial positions (%) |
|  | Years with female head of state (last 50), share of tenure years |
