# Supplementary material for: Gender equality related to gender differences in life expectancy across the globe gender equality and life expectancy
Source: PLOS Glob Public Health. 2023 Mar 6;3(3):e0001214. doi: 10.1371/journal.pgph.0001214 (PMC10021358; doi:10.1371/journal.pgph.0001214)
Supplement: S2 Table — (DOCX) [file pgph.0001214.s002.docx]

**S2 Table: Categorisation of countries into regions**

| Central Asia and Central and Eastern Europe | North Africa and Middle East | High-income countries | Latin America and Caribbean | South and Southeast Asia and Oceania | Sub-Saharan Africa |
| --- | --- | --- | --- | --- | --- |
| Albania | Algeria | Australia | Argentina | Afghanistan | Angola |
| Armenia | Bahrain | Austria | Bahamas | Bangladesh | Benin |
| Azerbaijan | Egypt | Belgium | Barbados | Bhutan | Botswana |
| Belarus | Iran | Canada | Belize | Brunei Darussalam | Burkina Faso |
| Bosnia and Herzegovina | Iraq | Cyprus | Bolivia | Cambodia | Burundi |
| Bulgaria | Jordan | Denmark | Brazil | China | Cabo Verde |
| Croatia | Kuwait | Finland | Chile | Fiji | Cameroon |
| Czech Republic | Lebanon | France | Colombia | India | Chad |
| Estonia | Morocco | Germany | Costa Rica | Indonesia | Cote d'Ivoire |
| Georgia | Oman | Greece | Cuba | Lao PDR | DR Congo |
| Hungary | Qatar | Iceland | Dominican Republic | Malaysia | Ethiopia |
| Kazakhstan | Saudi Arabia | Ireland | Ecuador | Maldives | Gambia |
| Kyrgyzstan | Syrian Arab Republic | Israel | El Salvador | Myanmar | Ghana |
| Latvia | Tunisia | Italy | Guatemala | Nepal | Guinea |
| Lithuania | Turkey | Japan | Guyana | Pakistan | Kenya |
| Macedonia (TFYR) | United Arab Emirates | Luxembourg | Honduras | Papua New Guinea | Lesotho |
| Moldova | Yemen | Malta | Jamaica | Philippines | Liberia |
| Mongolia |  | Netherlands | Mexico | Sri Lanka | Madagascar |
| Montenegro |  | New Zealand | Nicaragua | Thailand | Malawi |
| Poland |  | Norway | Panama | Timor-Leste | Mali |
| Romania |  | Portugal | Paraguay | Vanuatu | Mauritania |
| Russian Federation |  | Singapore | Peru | Viet Nam | Mauritius |
| Serbia |  | South Korea | Suriname |  | Mozambique |
| Slovakia |  | Spain | Trinidad and Tobago |  | Namibia |
| Slovenia |  | Sweden | Uruguay |  | Niger |
| Tajikistan |  | Switzerland | Venezuela |  | Nigeria |
| Ukraine |  | United Kingdom |  |  | Rwanda |
|  |  | United States of America |  |  | Senegal |
|  |  |  |  |  | Sierra Leone |
|  |  |  |  |  | South Africa |
|  |  |  |  |  | Swaziland |
|  |  |  |  |  | Tanzania |
|  |  |  |  |  | Togo |
|  |  |  |  |  | Uganda |
|  |  |  |  |  | Zambia |
|  |  |  |  |  | Zimbabwe |
