# Supplementary material for: Gender equality related to gender differences in life expectancy across the globe gender equality and life expectancy
Source: PLOS Glob Public Health. 2023 Mar 6;3(3):e0001214. doi: 10.1371/journal.pgph.0001214 (PMC10021358; doi:10.1371/journal.pgph.0001214)
Supplement: S3 Table — (DOCX) [file pgph.0001214.s003.docx]

**S3 Table: Association between change in the mGGGI and its subindexes and change in LE for women and men and gender gap in LE between 2010 and 2020**

|  | Estimate | 95CILB | 95CIUB | p-value |
| --- | --- | --- | --- | --- |
| mGGGI |  |  |  |  |
| Gender gap in LE | 0.00 | -0.29 | 0.30 | 0.985 |
| Women’s LE | -0.05 | -0.95 | 0.85 | 0.913 |
| Men’s LE | -0.05 | -1.08 | 0.99 | 0.928 |
| Economic subindex |  |  |  |  |
| Gender gap in LE | -0.12 | -0.29 | 0.04 | 0.138 |
| Women’s LE | -0.42 | -0.99 | 0.14 | 0.144 |
| Men’s LE | -0.30 | -0.80 | 0.20 | 0.238 |
| Education subindex |  |  |  |  |
| Gender gap in LE | 0.45 | 0.12 | 0.78 | <0.001 |
| Women’s LE | 2.51 | 1.41 | 3.61 | <0.001 |
| Men’s LE | 2.06 | 1.09 | 3.03 | <0.001 |
| Political subindex |  |  |  |  |
| Gender gap in LE | 0.02 | -0.13 | 0.17 | 0.784 |
| Women’s LE | -0.15 | -0.66 | 0.36 | 0.559 |
| Men’s LE | -0.17 | -0.62 | 0.27 | 0.448 |

Values represent the change in each outcome in years per 10% increase (i.e., greater gender equality) in each indicator.
