# Supplementary material for: Gender equality related to gender differences in life expectancy across the globe gender equality and life expectancy
Source: PLOS Glob Public Health. 2023 Mar 6;3(3):e0001214. doi: 10.1371/journal.pgph.0001214 (PMC10021358; doi:10.1371/journal.pgph.0001214)
Supplement: S4 Table — (DOCX) [file pgph.0001214.s004.docx]

**S4 Table: Cross-sectional association between the mGGGI and its subindexes and LE for women and men and gender gap in LE in 2021**

|  | Estimate | 95CILB | 95CIUB | p-value |
| --- | --- | --- | --- | --- |
| mGGGI |  |  |  |  |
| Gender gap in LE | 0.72 | 0.37 | 1.07 | <0.001 |
| Women’s LE | 3.65 | 2.48 | 4.82 | <0.001 |
| Men’s LE | 2.93 | 1.77 | 4.08 | <0.001 |
| Economic subindex |  |  |  |  |
| Gender gap in LE | 0.44 | 0.20 | 0.68 | <0.001 |
| Women’s LE | 0.44 | -0.45 | 1.34 | 0.330 |
| Men’s LE | 0.01 | -0.85 | 0.86 | 0.990 |
| Education subindex |  |  |  |  |
| Gender gap in LE | 6.09 | 5.08 | 7.11 | <0.001 |
| Women’s LE | 5.04 | 3.98 | 6.11 | <0.001 |
| Men’s LE | 1.05 | 0.69 | 1.41 | <0.001 |
| Political subindex |  |  |  |  |
| Gender gap in LE | 0.11 | -0.11 | 0.32 | 0.331 |
| Women’s LE | 1.56 | 0.84 | 2.27 | <0.001 |
| Men’s LE | 1.45 | 0.77 | 2.13 | <0.001 |

Values represent the change in each outcome in years per 10% increase (i.e., greater gender equality) in each indicator.
