# Supplementary material for: Gender equality related to gender differences in life expectancy across the globe gender equality and life expectancy
Source: PLOS Glob Public Health. 2023 Mar 6;3(3):e0001214. doi: 10.1371/journal.pgph.0001214 (PMC10021358; doi:10.1371/journal.pgph.0001214)
Supplement: S5 Table — (DOCX) [file pgph.0001214.s005.docx]

**S5 Table: Cross-sectional association between the mGGGI and LE for women and men and gender gap in LE stratified by region in 2021**

| Gender gap in life expectancy | Estimate | 95CILB | 95CIUB | p-value |
| --- | --- | --- | --- | --- |
| HIC | -0.51 | -0.95 | -0.07 | 0.032 |
| LAC | -0.71 | -2.11 | 0.69 | 0.332 |
| NAME | -0.70 | -1.99 | 0.60 | 0.307 |
| CACE | 0.77 | -0.83 | 2.36 | 0.355 |
| SSA | 1.36 | 0.63 | 2.09 | 0.001 |
| SEO | 1.13 | 0.29 | 1.96 | 0.015 |
| Women’s life expectancy |  |  |  |  |
| HIC | -0.44 | -1.12 | 0.23 | 0.211 |
| LAC | 1.50 | -0.91 | 3.92 | 0.233 |
| NAME | 3.55 | 1.66 | 5.44 | 0.002 |
| CACE | 1.92 | 0.01 | 3.84 | 0.060 |
| SSA | 3.04 | 1.01 | 5.08 | 0.006 |
| SEO | 2.28 | 0.23 | 4.33 | 0.042 |
| Men’s life expectancy |  |  |  |  |
| HIC | 0.07 | -0.69 | 0.82 | 0.860 |
| LAC | 2.21 | -0.65 | 5.07 | 0.143 |
| NAME | 4.25 | 2.30 | 6.20 | 0.001 |
| CACE | 1.16 | -1.54 | 3.85 | 0.408 |
| SSA | 1.68 | -0.17 | 3.54 | 0.084 |
| SEO | 1.15 | -0.76 | 3.05 | 0.252 |
