# Supplementary material for: Gender equality related to gender differences in life expectancy across the globe gender equality and life expectancy
Source: PLOS Glob Public Health. 2023 Mar 6;3(3):e0001214. doi: 10.1371/journal.pgph.0001214 (PMC10021358; doi:10.1371/journal.pgph.0001214)
Supplement: S6 Table — (DOCX) [file pgph.0001214.s006.docx]

**S6 Table: Cross-sectional association between the economic subindex of the mGGGI and LE for women and men and gender gap in LE stratified by region in 2021**

| Gender gap in life expectancy | Estimate | 95CILB | 95CIUB | p-value |
| --- | --- | --- | --- | --- |
| HIC | -0.70 | -1.24 | -0.16 | 0.018 |
| LAC | -0.87 | -1.58 | -0.15 | 0.026 |
| NAME | -0.82 | -1.70 | 0.07 | 0.092 |
| CACE | 1.51 | 0.53 | 2.48 | 0.006 |
| SSA | 0.23 | -0.43 | 0.88 | 0.506 |
| SEO | 0.53 | 0.10 | 0.96 | 0.027 |
| Women’s life expectancy |  |  |  |  |
| HIC | -0.67 | -1.50 | 0.16 | 0.124 |
| LAC | -0.04 | -1.42 | 1.34 | 0.953 |
| NAME | 2.38 | 0.91 | 3.85 | 0.006 |
| CACE | 0.61 | -0.82 | 2.03 | 0.414 |
| SSA | -0.01 | -1.77 | 1.74 | 0.987 |
| SEO | 0.83 | -0.27 | 1.92 | 0.155 |
| Men’s life expectancy |  |  |  |  |
| HIC | 0.02 | -0.92 | 0.97 | 0.960 |
| LAC | 0.82 | -0.81 | 2.46 | 0.333 |
| NAME | 3.20 | 1.83 | 4.56 | <0.001 |
| CACE | -0.90 | -2.79 | 0.99 | 0.358 |
| SSA | -0.24 | -1.73 | 1.25 | 0.754 |
| SEO | 0.30 | -0.69 | 1.29 | 0.561 |
