# Supplementary material for: Gender equality related to gender differences in life expectancy across the globe gender equality and life expectancy
Source: PLOS Glob Public Health. 2023 Mar 6;3(3):e0001214. doi: 10.1371/journal.pgph.0001214 (PMC10021358; doi:10.1371/journal.pgph.0001214)
Supplement: S7 Table — (DOCX) [file pgph.0001214.s007.docx]

**S7 Table: Cross-sectional association between the education subindex of the mGGGI and LE for women and men and gender gap in LE stratified by region in 2021**

| Gender gap in life expectancy | Estimate | 95CILB | 95CIUB | p-value |
| --- | --- | --- | --- | --- |
| HIC | -7.32 | -12.53 | -2.11 | 0.011 |
| LAC | -0.96 | -8.36 | 6.44 | 0.802 |
| NAME | -0.22 | -1.43 | 0.99 | 0.727 |
| CACE | 6.86 | 1.46 | 12.26 | 0.020 |
| SSA | 1.07 | 0.59 | 1.56 | <0.001 |
| SEO | 0.60 | -0.19 | 1.38 | 0.152 |
| Women’s life expectancy |  |  |  |  |
| HIC | -13.02 | -19.96 | -6.07 | 0.001 |
| LAC | 10.79 | -1.36 | 22.94 | 0.094 |
| NAME | 3.82 | 2.47 | 5.17 | <0.001 |
| CACE | 7.59 | 0.55 | 14.64 | 0.045 |
| SSA | 2.61 | 1.27 | 3.94 | 0.001 |
| SEO | 2.48 | 0.87 | 4.09 | 0.007 |
| Men’s life expectancy |  |  |  |  |
| HIC | -5.70 | -14.70 | 3.30 | 0.226 |
| LAC | 11.75 | -3.06 | 26.55 | 0.133 |
| NAME | 4.04 | 2.39 | 5.68 | <0.001 |
| CACE | 0.73 | -9.40 | 10.86 | 0.889 |
| SSA | 1.53 | 0.28 | 2.79 | 0.023 |
| SEO | 1.88 | 0.42 | 3.35 | 0.020 |
