# Supplementary material for: Gender equality related to gender differences in life expectancy across the globe gender equality and life expectancy
Source: PLOS Glob Public Health. 2023 Mar 6;3(3):e0001214. doi: 10.1371/journal.pgph.0001214 (PMC10021358; doi:10.1371/journal.pgph.0001214)
Supplement: S8 Table — (DOCX) [file pgph.0001214.s008.docx]

**S8 Table: Cross-sectional association between the political subindex of the mGGGI and LE for women and men and gender gap in LE stratified by region in 2021**

| Gender gap in life expectancy | Estimate | 95CILB | 95CIUB | p-value |
| --- | --- | --- | --- | --- |
| HIC | -0.19 | -0.39 | 0.00 | 0.064 |
| LAC | 0.06 | -0.37 | 0.50 | 0.771 |
| NAME | -0.14 | -1.07 | 0.79 | 0.767 |
| CACE | -0.26 | -0.99 | 0.47 | 0.498 |
| SSA | 0.50 | 0.03 | 0.96 | 0.044 |
| SEO | 0.27 | -0.43 | 0.97 | 0.459 |
| Women’s life expectancy |  |  |  |  |
| HIC | -0.15 | -0.44 | 0.15 | 0.330 |
| LAC | 0.39 | -0.35 | 1.12 | 0.312 |
| NAME | 0.73 | -1.04 | 2.50 | 0.434 |
| CACE | 0.82 | -0.06 | 1.70 | 0.078 |
| SSA | 1.26 | 0.03 | 2.49 | 0.053 |
| SEO | 0.11 | -1.57 | 1.78 | 0.903 |
| Men’s life expectancy |  |  |  |  |
| HIC | 0.04 | -0.28 | 0.37 | 0.801 |
| LAC | 0.32 | -0.57 | 1.22 | 0.487 |
| NAME | 0.87 | -1.10 | 2.84 | 0.399 |
| CACE | 1.08 | -0.09 | 2.24 | 0.082 |
| SSA | 0.77 | -0.31 | 1.84 | 0.174 |
| SEO | -0.17 | -1.61 | 1.28 | 0.825 |
