# Supplementary material for: Gender equality related to gender differences in life expectancy across the globe gender equality and life expectancy
Source: PLOS Glob Public Health. 2023 Mar 6;3(3):e0001214. doi: 10.1371/journal.pgph.0001214 (PMC10021358; doi:10.1371/journal.pgph.0001214)
Supplement: Attachment — Submitted filename: Response to Reviewers v2.docx [file pgph.0001214.s009.docx]

**Response to Reviewers**

**Reviewer #1:**

We thank Reviewer #1 very much for their comments.

- This is an important and outstanding topic of research. However, the novelty of the study was not highlighted! Adding the novelty of your research to the introduction section would underline its importance.

We added a paragraph to the introduction clarifying how our study builds on and expands previous research on this topic:

Page 4, line 85

In keeping with this, previous studies have suggested that gender equality may be associated with the gender gap in LE.[14, 15] Whilst in Europe and the Americas, gender equality appears to be associated with a wider gap in LE, in Africa gender equality may be associated with a narrower gap in LE. These studies used the United Nations Gender Inequality Index[14] and the European Union Gender Equality Index[15] to measure gender equality. However, there is no consensual method of measuring gender equality, and, despite some overlap, each index is based on different domains, which may have different associations with LE. For instance, the United Nations Gender Inequality Index comprises three dimensions: reproductive health (maternal mortality ratio; adolescent birth rate); empowerment (female and male population with at least secondary education; female and male shares of parliamentary seats); and labour market participation (female and male participation rates).[14] Although it is available for most countries in the world, data are lacking for some countries. Moreover, it provides a relatively simple and, hence, limited assessment of gender equality. Therefore, this study aimed to investigate to what extent gender equality, as measured by the comprehensive index developed by the World Economic Forum, was associated with the gender gap in LE across the globe.

- Data analysis on R is not available, making the results presented by the authors questionable, especially since the study shows outstanding results. For example: "there was no association between the change in the mGGGI and the change in the gender gap in LE."
We decided to provide the R code for the analysis and the necessary data are now available on GitHub (https://github.com/Ana-Catarina/global-gender-gap.git). All data are publicly available or provided in the manuscript and supplementary file. We added further details on the linear regression model and the R packages to the methods:

Page 5, line 146

Linear regression was used to estimate the association between change in the mGGGI, and its subindices, and change in the gender gap in LE, LE for women and LE for men between 2010 and 2020. Linear regression was also used to estimate the cross-sectional association between the mGGGI, and its subindices, in 2021 and the gender gap in LE, LE for women and LE for men using the most recent data (from 2020). Simple linear regression models were used with no adjustments. Analyses were performed separately for all subgroups. Gender gap indices with 95% confidence intervals (CI) were reported and displayed using scatter plots. All analyses were carried out using the base package and all figures constructed using the ggplot2 package for R version 4.1.2. All the code and data needed to replicate this analysis is publicly available on a repository (https://github.com/Ana-Catarina/global-gender-gap.git).

- I would recommend discussing the definition of the "gender equality" term and how it might be related to LE according to the literature.
We added a paragraph to the introduction discussing the definition of gender equality:

Page 3, line 52

Notwithstanding substantial progress made over the past centuries, gender equality in all spheres of private and societal life remains far off and fulfilment of women’s rights is lagging worldwide.[1] Gender equality implies that the interests, needs and priorities of both women and men are taken into consideration, thereby recognising the diversity of different groups of women and men.[2] It does not mean that women and men will become the same but that women’s and men’s rights, responsibilities and opportunities will not depend on whether they are born female or male. According to the United Nations, equality between women and men is seen both as a human rights issue and as a precondition for, and indicator of, sustainable people-centred development.[3]

- The literature review is missing, which is essential to understand the reason behind the adapted methods and methodology in this study. How would we know that the applied methodology is appropriate for this type of study without a relevant literature review?

We carried out a literature review and previous studies are discussed in the introduction and discussion. We adopted a conventional and well-established methodology to investigate potential associations at population level (i.e., this is a typical ecological study). We added references to similar studies on this topic to the methods:

Page 4, line 105

We adopted a similar methodology to previous studies on this topic.[14-16]

- More discussion is required for the Study Design section. What is an ecological study? It seems more like a description of the study than about methodology.

An ecological study is a clearly defined epidemiological concept and type of study. An ecological study assesses the overall frequency of disease in a series of populations and looks for a correlation with the average exposure in the populations. These studies do not use data on individuals. Instead, the data points are the average levels of exposure and the overall frequency of disease in a series of populations. Therefore, the unit of observation is not a person; rather, it is an entire population or group. To help readers who may be unfamiliar with epidemiology, we added a sentence to the study design:

Page 4, line 103

We conducted an ecological study to investigate the association between gender equality and the gender gap in LE in worldwide. In this ecological study, the unit of analysis was the population of each country.

- More discussion is required on the four sub-indices and how changes in these sub-indices are reflected on GGGI. The authors mentioned in the Discussion section, "which is mainly driven by its political and economic subindexes as the education subindex remained relatively stable", which means that the discussion is related to how these sub-indices change the GGGI.
The indices are all weighted equally on the mGGGI, which means that changes in any of the subindices will be reflected similarly on the mGGGI. We make this point clear in the methods by stating that the GGGI is a simple average of the four subindices. The aim of this study is to investigate the association between the mGGGI and its subindices and LE and the gender gap in LE. Exploring the underlying trends on each of the subindices is not within the scope of this study and is well documented in the annual report published by the World Economic Forum, which we cite throughout the manuscript.

- Ref 10: could you please refer to the page number in the attributed reference? I tried to explore more information about the GGGI in the reference, but it was an uneasy task to refer directly to the target section in the reference, which would be the same for the readers.
Reference 10 in the original manuscript was

[10] Zarulli V, Kashnitsky I, Vaupel JW. Death rates at specific life stages mold the sex gap in life expectancy. Proceedings of the National Academy of Sciences. 2021;118(20):e2010588118. doi: doi:10.1073/pnas.2010588118.

There is no mention of GGGI in this reference.

- Gender equality section: "The MGGG for 2010 to 2020 .... evaluating time trends over a decade." there is no reference.
We added a reference to the World Economic Forum webpage where all the reports are available for consultation and download. These reports include data for the GGGI and LE for women and men.

Page 5, line 122

Data for the years 2006 to 2010 were excluded as estimates were missing for 42 out of 156 countries in 2006 but only for 23 countries in 2010 and this would allow evaluating time trends over a decade.[18]

- LE data are missing (for women, men, and the gender gap in LE calculation).
These data were obtained from the World Bank, which is a publicly available resource where readers can consult trends in LE for women and men for all the countries included in this study. Including these data as supplementary material would require including large tables, which would be difficult to read. The interactive website of the World Bank, which readers can access via the link in the reference, is a much better tool to understand trends in LE. In addition, the data are available on a GitHub repository (<https://github.com/Ana-Catarina/global-gender-gap.git>). We, therefore, decided it would be unnecessary to provide LE data as supplementary files.

Page 5, line 153

All the code and data needed to replicate this analysis are publicly available on a repository (https://github.com/Ana-Catarina/global-gender-gap.git).

- Life Expectancy section: "Data for LE at birth between .... from the World Bank. Data for LE in 2021 were not available" there is no reference.
We added a reference to the World Bank webpage from where data for LE were obtained.

Page 5, line 127

Data for LE at birth between 2010 and 2020 were obtained for women and men from the World Bank.[18] Data for LE in 2021 were not available when this study was performed.

- It seems like there is missing information before the Data Analysis section. The authors mentioned that the mGGGI and its sub-indices were converted into percentages. What are the data that they were converted? Where can the reader find them?
These data were directly retrieved from the annual Global Gender Gap reports published by the World Economic Forum, which are publicly available from the reference (website) cited in the methods. As explained in the methods, the GGGI measures a gender gap in proportion of representation, which means it ranges between 0 (imparity) and 1 (parity). As percentages are easier to understand than proportions, we converted the index and its subdomains, which are also calculated as proportions, into percentages by multiplying by 100. This is a simple calculation and we explain it in the methods. There is no information missing.

- I would recommend moving lines 103 to 110 to the Study Design section, where more discussion about the research methods and variables is required.
We have added a clarification to the study design section (please see above). The content of lines 103 to 110 pertains to data analysis, not study design.

- Data of the mGGGI and its sub-indices after converting into percentages and standardizing them are missing.
These data are publicly available on the annual reports published by the World Economic Forum and are displayed in Figure 1. The tables with all these data are too large to be readable as a word document. The data are available on a public GitHub repository (<https://github.com/Ana-Catarina/global-gender-gap.git>). We considered it would not add value to include them in the supplementary file.

Page 5, line 153

All the code and data needed to replicate this analysis are publicly available on a repository (<https://github.com/Ana-Catarina/global-gender-gap.git>).

- The categorization of the countries into regions is not referenced.
We added a reference to one of the GBD studies where the categorisation of countries into regions is mentioned:

Page 5, line 139

This classification based on geography and development employs the classification used by the Global Burden of Disease.[12]

- Why do the authors specifically use the Global Burden of Diseases categorization of countries?
As there is no standard categorisation of countries into regions, we decided to use the GBD categorisation because it combines geography and socioeconomic development, which are both likely to be related to LE and gender equality. Adopting a purely geographical classification, such as that employed by the World Economic Forum or the World Health Organisation, would group together countries with very different stages of development and gender equality policies. Among all the possible classifications, the GBD appeared to be the most balanced and appropriate for this study. We explained this in the methods:

Page 5, line 139

This classification based on geography and development employs the classification used by the Global Burden of Disease.[12] As there is no standard classification of countries into regions, this appeared to be the most appropriate approach because LE and gender equality are likely to be influenced by both socioeconomic development and geographical proximity. Using a purely geographical classification would result in significant heterogeneity within categories, which would render regional measures misleading and bias the association towards the null.

- The method the results were presented is inconsistent and confusing. I would suggest rephrasing this section.
We are sorry that the reviewer found the results inconsistent and confusing. There are many results to present and we considered this was the best way to present them.

- In the Discussion section, the authors justified the discrepancy by the fact that there are several HIC in the region of Asia and Oceania. However, referring back to the categorization of the countries in the Data Analysis section, I would presume that HIC is a separate group in terms of data analysis.
We apologise for the confusion. The sentence mentioned by the reviewer refers to a previous study that conducted similar analysis but using the United Nations. As we explain in the methods and discussion, this purely geographical classification may be inadequate for the purpose of investigating the association between gender equality and the gender gap in LE because countries in geographical proximity may be at different stages of socioeconomic development, which is related to gender equality. We clarified this in the discussion:

Page 8, line 255

This discrepancy may be due to the fact that the previous study adopted the United Nations classification of countries into pure geographical regions.[16] In consequence, Asia and Oceania included several HIC and the resulting heterogeneity within these regions would dilute a potential association.

- Lines 231 to 278 sound irrelevant to the Discussion section. The authors presenting subjective opinions about the importance of the study. This paragraph seems to be more about the introduction of the study after major rephrasing.
Although we understand reviewer’s point about subjectivity, we believe this section is a key component of the discussion as it discusses the implications of our findings for policy across multiple sectors of government and society in general. We don’t agree this section could be included in the introduction as it discusses the implications of our findings, which are only presented in the results. The second reviewer also considered this section to be relevant and interesting. Therefore, we decided to keep it.

- The reason behind categorizing the countries into multiple groups is mentioned in the Discussion section. I would recommend moving it to the method section.

The rationale for the categorisation of countries into groups based on socioeconomic development and geographic location is well explained in the methods:

Page 5, line 139

This classification based on geography and development employs the classification used by the Global Burden of Disease.[12] As there is no standard classification of countries into regions, this appeared to be the most appropriate approach because LE and gender equality are likely to be influenced by both socioeconomic development and geographical proximity. Using a purely geographical classification would result in significant heterogeneity within categories, which would render regional measures misleading and bias the association towards the null.

**Reviewer #2:**

We thank Reviewer #2 very much for their comments.

The research study investigated the association of gender equality with the gender gap in life expectancy (LE) across the globe. The study uses a modified version of the Global Gender Gap Index - excluding the health subindex because it included life expectancy. The longitudinal analyses did not find an association between the change in the mGGGI and the change in the gender gap in LE. There was also no association between the change in the mGGGI and the change in LE for women and men. While there was no association between the change in the economic and political subindexes and changes in the LE gender gap or LE for women and men, there was a widening of the LE gender gap for the education subindex. There were some more associations found in the cross-sectional analyses. These were explained well in the discussion section. It would be helpful if authors clarified what an "increase in mGGGI" means. This research study has great potential for publication. There are some minor edits for consideration in order to strengthen and tighten the reporting of the study.

One, it would be helpful to clarify in which direction the gender gap is widening, throughout the manuscript. It is my understanding that there are several ways the gender gap can increase: increase in LE for women while it remains the same for men; decrease in LE for men, while staying the same for women; an increase in LE for men and women, with a greater increase for women; or an increase for women and a decrease for men. It would be helpful to clarify the reason for each difference in the gap whenever it is mentioned throughout the manuscript, or clarify terminology so that the reader can understand as they are reading.
We agree with this point and that is why we performed separate analysis for LE for women and men in addition to the analysis of the gender gap itself. In the results, whenever the gender gap is mentioned, this is preceded or followed by a description of the association for LE for women and en in isolation. As LE increased for women and men throughout the study period, changes in the gender gap were driven by larger or smaller increases in LE for women and men. We clarified the meaning of widening/increasing and narrowing/decreasing the gender gap in LE in the methods. We also clarified the drivers for the widening or narrowing of the gender gap in LE in the discussion.

Page 5, line 132

As LE increased for both women and men throughout the study period, the gender gap in LE was calculated as the difference (values in 2020 minus values in 2010) in LE between women and men for each country. A widening or increase in the gender gap in LE meant that LE increased more for women than men, whilst a narrowing or decrease in the gender gap in LE meant that LE increased more for men than women.

Page 8, line 232

Only improvements in gender equality in education were associated with a longer LE for women and men and widening of the gender gap in LE, due to a larger increase in LE for women than men.

The authors may want to consider linking the ideas in the introduction more with each other in order to develop the introduction further. Additionally, the authors may want to include references in the introduction that explain life expectancy and its role as a measure in public health from larger institutions (WHO, WEF, etc.) in addition to the citations already included.
We added a few paragraphs to the introduction and included two new references related to life expectancy as an indicator of population health.

References:

4.	Luy M, Di Giulio P, Di Lego V, Lazarevič P, Sauerberg M. Life Expectancy: Frequently Used, but Hardly Understood. Gerontology. 2020;66(1):95-104. doi: 10.1159/000500955.

5.	Ilknur K, Bahar S, Ozalp V. Analysis of life expectancy across countries using a decision tree. Eastern Mediterranean Health Journal. 2020;26(2):143-51. doi: <https://doi.org/10.26719/2020.26.2.143>.

The authors may want to write that there is no statistically significant association when writing in the results section, rather than that there is no association, wherever applicable.

We amended all the entries of “no association” to “no statistically significant” association in the results.

I'm not sure whether this study could be replicated based on the information that is provided in the methods section. The authors may want to consider providing more details about the setup of the linear regression models.
We provided additional details on the linear regression models in the methods and also shared all the code and the data required to replicate the analysis on a public GitHub repository (https://github.com/Ana-Catarina/global-gender-gap.git).

Page 5, line 146

Linear regression was used to estimate the association between change in the mGGGI, and its subindices, and change in the gender gap in LE, LE for women and LE for men between 2010 and 2020. Linear regression was also used to estimate the cross-sectional association between the mGGGI, and its subindices, in 2021 and the gender gap in LE, LE for women and LE for men using the most recent data (from 2020). Simple linear regression models were used with no adjustments. Analyses were performed separately for all subgroups. Gender gap indices with 95% confidence intervals (CI) were reported and displayed using scatter plots. All analyses were carried out using the base package and all figures constructed using the ggplot2 package for R version 4.1.2. All the code and data needed to replicate this analysis is publicly available on a repository (https://github.com/Ana-Catarina/global-gender-gap.git).

The discussion section is well-developed and explains the results well. The parts of the discussion with the policy recommendations would be made stronger if it included more support from literature. When writing recommendations such as "investing in education is paramount", "this is particularly important in LMIC", and suggestions for parental leave and flexible hours, the statements would be made even stronger if they had support from existing literature. Each recommendation and statement that includes information outside of the study should be supported by literature.

We added references to the recommendations, as suggested.

In the discussion section, the "lack of association between gender equality in the political subindex and the gender gap in LE" and the implications for policy was a little confusing; I would suggest making that connection more clear.

We amended that paragraph as follows:

Page 10, line 315

Fifth, the lack of association between gender equality in the political subindex and the gender gap in LE raises concerns about the extent to which tokenistic approaches to gender equality are being implemented by political systems worldwide. Gender quotas, although instrumental to change societal values and encourage women’s participation in politics, have not yet achieved gender parity in political parties and parliaments, even in HIC.[32] Furthermore, even in political organisations where women are present in large numbers, glass ceilings often remain firmly in place, thus limiting women’s power and ability to influence policy. Women still experience significant challenges as politicians, such as addressing discrimination or cultural beliefs that limit women’s role in society; balancing private, family and political life; gaining support from political parties; and securing campaign funding.[33] They may also face violence, harassment, and intimidation or be dissuaded from running for office.[33] Ultimately, this means that women still lack the power to influence policy and decision making even when numerically represented in political systems, which might explain we found no association between gender equality in politics and LE.

Given that the mGGGI includes economic opportunity, I'm not sure why one of the included limitations is that the observed associations between gender equality and HLE and LE are explained by other economic factors, even if the analysis didn't suggest it. This may be a major limitation.
The point we tried to make was that the association between gender equality and LE could depend on the level of socioeconomic development at country level rather than gender equality on the economic domain. As countries become more socioeconomically developed, it is likely that populations will live longer (i.e., longer LE) and societies will gradually move towards greater gender equality. However, this is by no means guaranteed as women may continue to be discriminated against and denied equal rights to men to sociocultural norms, principles and beliefs, often linked to religion. We amended the sentence to reflect these points:

Page 11, line 358

Second, it is possible the observed associations between gender equality and LE are explained by other factors, such as socioeconomic development, sociocultural norms or other unmeasured confounders.

The conclusion section makes statements that are not mentioned in the literature (i.e. ideal measures and markers for gender equality and rate of progress in the past decade) so should focus more on the findings and literature that are mentioned in the manuscript, or include this evidence in the introduction or the discussion. It would also be best to omit any informal language such as "long way off" and use technical language (that is still accessible for people to understand).

We amended the conclusion to reflect only the findings of our study:

Page 11, line 365

Globally, greater gender equality is associated with longer LE for both women and men. Greater gender equality is also associated with a widening of the gender gap in LE, due to a larger increase in LE for women than men. The variation in this association across world regions defined on socioeconomic development and geographic proximity suggests that gender equality may initially widen the gender gap in LE as the benefits of greater gender equality benefit mainly women’s lives and health. As countries progress along the continuum of gender equality, the benefits of increased participation of women in society extend to men, thus leading to a larger increase in men’s LE and a narrowing of the gender gap in LE. Therefore, addressing longstanding gender inequality and empowering women might help extending longevity for both women and men.

The figures should show the magnitude of the association within the figure.
We added the magnitude of the association and respective p-value to the legends of figures 3 and 4.

In general, there were a few typos throughout the manuscript, so the authors should check for grammar, typos, and clarity throughout the manuscript.
We revised the manuscript for clarity and correctness.

Overall, this is a very important topic and I am glad that you all undertook this study. Thank you for sharing this research!

Thank you very much.
